# Effect of radiotherapy on activity and concentration of serum paraoxonase-1 in breast cancer patients

**DOI:** 10.1371/journal.pone.0188633

**Published:** 2017-11-27

**Authors:** Meritxell Arenas, Anabel García-Heredia, Noemí Cabré, Fedra Luciano-Mateo, Anna Hernández-Aguilera, Sebastià Sabater, Marta Bonet, Marina Gascón, Salvador Fernández-Arroyo, Isabel Fort-Gallifa, Jordi Camps, Jorge Joven

**Affiliations:** 1 Department of Radiation Oncology, Hospital Universitari de Sant Joan, Institut d’Investigació Sanitària Pere Virgili (IISPV), Universitat Rovira i Virgili, Reus, Spain; 2 Unitat de Recerca Biomèdica (CRB-URB), Hospital Universitari de Sant Joan, Institut d’Investigació Sanitària Pere Virgili (IISPV), Universitat Rovira i Virgili, Reus, Spain; University of South Alabama Mitchell Cancer Institute, UNITED STATES

## Abstract

Paraoxonase-1 (PON1) is an intra-cellular antioxidant enzyme found also in the circulation associated with high-density lipoproteins. The activity of this enzyme has been shown to be decreased in breast cancer (BC) patients. The aims of our study were to investigate the changes produced by radiotherapy (RT) on activity and concentration of serum PON1 in BC patients, and to evaluate the observed variations in relation to clinical and pathological characteristics of patients and tumors, and the response to treatment. We studied 200 women with BC who were scheduled to receive RT following excision of the tumor. Blood for analyses was obtained before and after the irradiation procedure. The control group was composed of 200 healthy women. Relative to control, BC patients had significantly lower serum PON1 activities pre-RT, while PON1 concentrations were at similar levels. RT was associated with a significant increase in serum PON1 activities and concentrations. We observed significant differences in serum PON1 concentrations post-RT between patients with luminal A or luminal B tumors. Serum PON1 concentration post-RT was markedly lower in BC patients with metastases. We conclude that benefit from RT accrues to the BC patients not only through its direct effect on cancer cells but also indirectly by improving the organism’s anti-oxidant defense mechanisms. In addition, our preliminary evidence suggests that the measurement of serum PON1 concentration post-RT could be an efficient prognostic biomarker, and may be used as an index of the efficacy of the RT.

## Introduction

Breast cancer (BC) is the malignant disease with the highest incidence worldwide, and is the most common cancer-related cause of death in women [1]. Genetic factors, lifestyle, and environmental agents are important contributors to the etiology of BC but, nevertheless, the molecular mechanisms underlying BC onset and development have not, as yet, been elucidated [2]. For example, oxidative stress and lipid peroxidation products play a role in oncogenesis [3] while oxidized low-density lipoproteins (LDL) have been reported as being causally associated with oxidative stress-related cancers [4]. Recent investigations have suggested that paraoxonase-1 (PON1) plays a significant role in the molecular disorders associated with cancer. PON1 is an antioxidant enzyme found in the membranes of most cells and, as well, in the circulation bound to HDL [5–7]. The original function attributed to PON1 is that of a lactonase since lipophilic lactones constitute its primary substrates [8]. It is this catalytic capacity that enables PON1 to degrade lipid peroxides within the cell and in the lipoproteins in circulation [9]. In addition, PON1 has an esterase activity and degrades organophosphate xenobiotics such as paraoxon, phenylacetate and nerve agents [7]. PON1 levels are genetically determined; the polymorphisms Arg/Gln at position 192 (*PON1*_*192*_, with two alleles termed Q and R), and Leu/Met at position 55 (*PON1*_*55*_, with two alleles termed L and M) being strongly associated with the enzyme’s activity. *PON1*_*55*_ polymorphism indirectly influences serum PON1 activity and concentration i.e. it is in linkage disequilibrium with the *PON1*_*-108*_ promoter polymorphism; the mutation which is more clearly responsible for the observed changes [7]. Recent studies reported a decrease in serum PON1 activity in BC patients [2,10]. In addition, the M allele of the L55M polymorphism was associated with a higher incidence of BC [11–13].

The current preferred treatment of BC is surgery followed by loco-regional radiotherapy (RT) and, often, adjuvant chemotherapy and/or hormone therapy [14]. RT is an effective treatment but response is very variable and reflects the heterogeneity of the disease and its sensitivity to treatment. The efficacy of RT is reflected in the molecular classification of BC tumors; a classification which is based on the expression of certain proteins by the tumor cells. For example, the Her2 subtype has a positive expression of human epidermal growth factor receptor 2; the luminal A subtype has positive expression of hormone receptors (estrogens and/or progesterone), is Her2 negative and has an expression of the nuclear regulator of the cell proliferation Ki67 of <14%; the luminal B subtype is positive for the hormone and Her2 receptors and has a Ki67 expression >14%; the triple negative subtype is negative for hormone receptors and Her2. Luminal subtypes are associated with a more favorable prognosis, and are more sensitive to ionizing radiation [15,16].

The aims of our study were: 1) to investigate the changes produced by RT on activity and concentration of serum PON1 in BC patients; 2) to assess the possible influence of *PON1*_*192*_ and *PON1*_*55*_ polymorphisms; 3) to relate these changes with the clinical and pathological characteristics of the patients and their tumors, especially with respect to response-to-treatment.

## Methods

### Patients and samples

We included 200 women (mean age: 54 years, range: 37–84) diagnosed as having BC and attending the Department of Radiation Oncology of our Hospital after having undergone surgery for tumor extirpation. All patients had a Karnofsky Index > 70 and were classified as 0 or 1 on the Eastern Cooperative Oncology Group scale [17]. The exclusion criteria included: having previously received RT at the same cancer site, and being pregnant or lactating. Six patients (3%) had metastases. Based on studies suggesting benefit in this subgroup of patients [18], the protocol of our Center indicates post-operative RT in metastatic BC patients following pathological complete response of the metastases to primary systemic treatment.

In the present study, 33 patients received adjuvant chemotherapy post-surgery, 80 patients received adjuvant hormone therapy post-surgery, and 87 received both treatments. The adjuvant chemotherapy treatment duration was for about 4 to 5 months, and concluded 1 to 2 months before RT commencement. The adjuvant hormone treatment commenced 1 to 2 months post-surgery and, usually, was administered simultaneously with RT. The radiation schedule was normofractionated RT (50 Gy at 2 Gy/day on the breast and 16 Gy at 2 Gy/day on the tumor bed, 5 days/week) or hypofractionated RT (40 Gy at 2.67 Gy/day, 5 days/week) [19,20]. Thirty percent of the patients received irradiation of regional lymph nodes, according to existing risk factors [21,22]. During RT, a weekly acute toxicity assessment was performed using the criteria of the Radiation Therapy Oncology Group and the European Organization for Research and Treatment of Cancer [23].

Prior to irradiation, and one month after completion of RT, blood samples were obtained and sera and leukocytes were stored at– 80°C until processed for biochemical and genetic analyses. The control group was composed of 200 healthy women (mean age: 47 years, range: 33–84) participating in a population-based study conducted in our geographical area. They had no clinical or analytical evidence of infectious disease, renal insufficiency, hepatic damage, neoplasia, oligophrenia, or dementia. A detailed description of this population has been published recently [24]. All patients and control subjects signed a written informed consent according to the declaration of Helsinki. The study was approved by the Ethics Committee (Institutional Review Board) of the *Hospital Universitari de Sant Joan* (project code: 14/2017).

### Biochemical measurements

Serum PON1 paraoxonase activity was determined as the rate of hydrolysis of paraoxon at 410 nm and 37°C in a 0.05 mM glycine buffer, pH 10.5 with 1 mM CaCl_2_ [25]. Activities were expressed as U/L (1 U = 1 μmol of paraoxon hydrolyzed per minute). Serum PON1 concentrations were determined by in-house ELISA with rabbit polyclonal antibodies generated against the synthetic peptide CRNHQSSYQTRLNALREVQ which is a sequence specific for mature PON1 [26,27]. Serum alanine aminotransferase (ALT) and aspartate amino transferase (AST) activities, and lipid profile were analyzed by standard tests in a Roche Modular Analytics P800 system (Roche Diagnostics, Basel, Switzerland).

### PON1 genotyping

Genomic DNA was obtained from leukocytes (Puregene DNA Isolation reagent set, Gentra Systems Inc., Minneapolis, MN). *PON1*_*192*_ and *PON1*_*55*_ single nucleotide polymorphisms were analyzed with GoTaq^®^ qPCR Master Mix reagents (Promega Corp., Madison, WI) in a 7900HT Fast Real-Time PCR System (Applied Biosystems, Foster City, CA).

### Statistical analysis

All the calculations were performed with SPSS 22.0 statistical package (SPSS Inc., Chicago, IL, USA). Since PON1-related variables present with non-Gaussian distributions, differences between any two groups were assessed with the Mann-Whitney *U* test. Spearman correlation coefficients were used to evaluate the degree of association between quantitative variables. The χ-square test was employed to evaluate differences in qualitative variables. Linear regression analysis was employed to investigate the independent association between selected variables and serum PON1 activity and concentration in the overall population. Receiver operating characteristics (ROC) curve analyses were employed to evaluate the diagnostic accuracy of the measured biochemical variables. This analysis employs plots of different sensitivity/specificity pairs based on varying decision thresholds. Sensitivity (or true positive rate) is the proportion of the sample correctly identified as associating with a specific disease. Specificity (or true negative rate) is the proportion of subjects correctly identified as not segregating with a specific disease. False positive rate is calculated as 1-specificity. The area under the curve (AUC) and 95% confidence interval (CI) values are calculated. The AUC represents the ability of the test to correctly classify patients with the alteration being investigated. The values of AUC can range between 1 (perfect test) and 0.5 (worthless test) [28]. Results are shown as medians and 95%CI.

## Results

### Clinical characteristics of patients

The main clinical characteristics of the BC patients and their tumors are shown in Table 1. Patients attending the Department of Radiation Oncology already have their tumors operated upon. In the majority of cases the tumors were classified as luminal A or B, were positive for estrogen and progesterone receptors, were relatively small in size and had not developed metastases. The most common pathological diagnosis was that of invasive ductal carcinoma. Individual results of all the clinical, demographic and analytical variables are shown in the Supporting Information (S1 Table).

**Table 1:** Clinical characteristics of the breast cancer patients, tumors treated and surgical procedures.

Variable	Frequency (%) n = 200
*Smoking*	25.0
*Alcohol habit (> 20g/day)*	4.5
*Arterial hypertension*	28.0
*Diabetes*	8.5
*Dyslipidemia*	27.5
*Chronic obstructive pulmonary disease*	4.5
*Ischemic heart disease*	3.5
*Hypothyroidism*	9.0
*Menopause state*	
Premenopausal	24.5
Perimenopausal	12.0
Postmenopausal	63.5
*Intake of oral contraceptives*	30.5
*Tumor size (TNM system)*	
T1	63.0
T2	28.5
T3	6.5
T4	2.0
*Nodes (TNM system)*	
N0	68.0
N1	24.5
N2	6.0
N3	1.5
*Metastases (TNM system)*	
M0	97.0
M1	3.0
*Pathological anatomy of the tumor*	
Ductal carcinoma in situ	7.5
Invasive ductal carcinoma	79.0
Lobular carcinoma in situ	0.5
Invasive lobular carcinoma	1.5
Papillary carcinoma	6.5
Others	5.0
*Estrogen receptors*	
Negative	17.0
Positive	83.0
*Progesterone receptors*	
Negative	32.0
Positive	68.0
*Ki67 antigen in tumor biopsy*	
Less than 15%	42.5
15–50%	44.5
More than 50%	13.0
*Her2 receptor in tumor biopsy*	
Negative	81.0
Positive	19.0
*Tumor molecular classification*	
Luminal A	34.0
Luminal B	36.5
Her2 positive	19.0
Triple negative	10.5
*Surgical procedure*	
Lumpectomy	79.0
Mastectomy	21.0
*Adjuvant chemotherapy*	60.0
*Adjuvant hormone therapy*	83.5

### Changes in biochemical variables

Relative to control subjects, BC patients had significantly lower serum PON1 activities pre-RT, while PON1 concentrations were similar. They also had similar AST and ALT activities, and higher cholesterol and triglyceride concentrations. RT was associated with significant increases in serum PON1 activities and concentrations while other analyzed variables were not significantly altered (Table 2). Linear regression analyses showed that BC, together with *PON1*_*192*_ and *PON1*_*55*_ polymorphisms, were independent predictors of serum PON1 activity (but not of PON1 concentration). Age was not significantly associated with these variables (Table 3).

**Table 2:** Results of the selected biochemical variables in control women (n = 200) and in breast cancer (BC) patients (n = 200) pre- and post-radiotherapy.

Variable	Control	BC Pre-RT	BC Post-RT	*P*-value Control *vs*. BC Pre-RT	*P*-value Control *vs*. BC Post-RT	*P*-value BC Pre- *vs*. Post-RT
*AST (μkat/L)*	0.33 (0.25–0.57)	0.32 (0.22–0.54)	0.33 (0.20–0.53)	0.181	0.776	0.381
*ALT (μkat/L)*	0.27 (0.15–0.67)	0.31 (0.17–0.67)	0.31 (0.17–0.62)	0.031	0.131	0.429
*Cholesterol (mmol/L)*	5.10 (3.60–7.00)	5.68 (3.94–7.28)	5.50 (3.99–7.56)	< 0.001	0.008	0.146
*HDL cholesterol (mmol/L)*	1.54 (0.98–2.29)	1.56 (0.99–2.34)	1.55 (1.00–2.18)	0.173	0.537	0.447
*LDL cholesterol (mmol/L)*	3.07 (1.65–4.88)	3.21 (1.93–4.68)	3.12 (1.61–4.77)	0.147	0.882	0.238
*VLDL cholesterol (mmol/L)*	0.45 (0.23–1.04)	0.65 (0.33–1.28)	0.62 (0.29–1.53)	< 0.001	< 0.001	0.611
*Triglycerides (mmol/L)*	1.00 (0.50–2.20)	1.34 (0.69–3.48)	1.41 (0.69–3.03)	< 0.001	< 0.001	0.965
*PON1 activity (U/L)*	274.3 (156.8–565.9)	154.9 (99.9–260.2)	170.3 (97.2–400.1)	< 0.001	< 0.001	0.040
*PON1 concentration (mg/L)*	97.3 (43.2–285.3)	91.7 (30.6–223.3)	111.8 (23.7–245.0)	0.081	0.308	0.018

ALT, alanine aminotransferase; AST, aspartate aminotransferase, HDL, high-density lipoproteins; LDL, low-density lipoproteins; PON1, paraoxonase-1; VLDL, very low-density lipoproteins. Results are shown as medians and 95% Confidence Interval (in parenthesis).

**Table 3:** Linear regression analyses of the variables independently associated with PON1 activity and concentration in breast cancer patients and the control group of subjects.

PON1 activity; U/L*	B	95% CI of B	*P*
Constant	32.317	-9.215–73.850	0.127
Population	133.856	124.814–152.897	< 0.001
Age	-0.075	-0.517–0.367	0.739
*PON1*_*55*_	-13.997	-24.909–3.086	0.012
*PON1*_*192*_	107.209	95.192–119.226	< .001
*PON1 concentration; mg/L* †			
Constant	94.457	52.414–136.501	< 0.001
Population	11.474	-2.701–25.649	0.112
Age	-0.122	-0.567–0.323	0.590
*PON1*_*55*_	1.723	-9.323–12.769	0.759
*PON1*_*192*_	10.396	-1.669–22.461	0.091

*Model summary: *r*^*2*^ = 0.696; *P* < 0.001;

^†^ Model summary: *r*^*2*^ = 0.017; *P* = 0.134.

Population: Breast cancer = 0; Control group = 1. B: Unstandardized regression coefficient. *P* is the probability value that each variable included in the model is independently associated with PON1 values.

### Lack of relationship between *PON1* genotypes and BC

The allele distributions of the *PON1*_*192*_ and *PON1*_*55*_ polymorphisms followed Hardy-Weinberg equilibrium in cases as well as in control subjects. There were no significant differences in the genotype frequencies of the analyzed *PON1* gene polymorphisms between women with BC and the healthy control individuals (Table 4).

**Table 4:** Genotype frequencies (%) of the analyzed PON1 gene polymorphisms in patients with breast cancer and in the control group of subjects. Differences were assessed with the χ-square test.

*PON1*_*55*_	**LL**		**LM**		**MM**		***P*-value**
	*Control group*	*Breast cancer*	*Control group*	*Breast cancer*	*Control group*	*Breast cancer*	
	33.0	39.0	42.1	48.1	18.5	15.8	0.182
*PON1*_*192*_	**QQ**		**QR**		**RR**		***P*-value**
	*Control group*	*Breast cancer*	*Control group*	*Breast cancer*	*Control group*	*Breast cancer*	
	51.1	55.9	40.8	34.9	8.1	9.2	0.456

**Fig 1:**
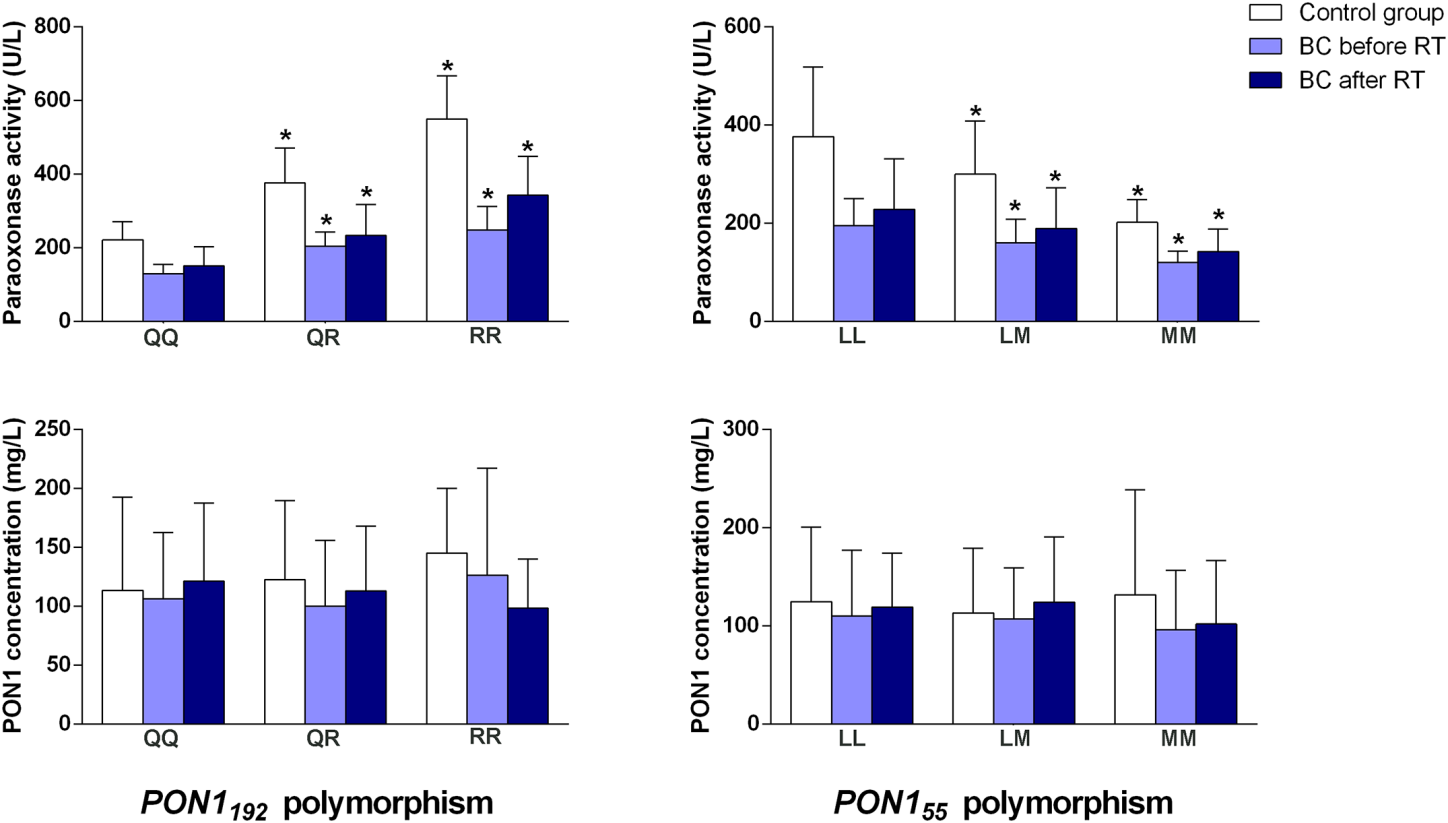
Serum paraoxonase (PON1) activities and concentrations pre- and post-radiotherapy (RT) in patients with breast cancer (BC) segregated with respect to the investigated *PON1* gene polymorphisms. *: *P* < 0.05 with respect to the wild-type isoform (QQ and LL, respectively).

When we segregated all the subjects according to the *PON1* genotypes, we observed that, relative to control subjects the degree of decrease of PON1 activity in BC patients pre-RT as well as the degree of increase post-RT were similar for each of the isoforms (Fig 1).

### Relationships between PON1-related variables and the patients’ clinical characteristics

**Fig 2:**
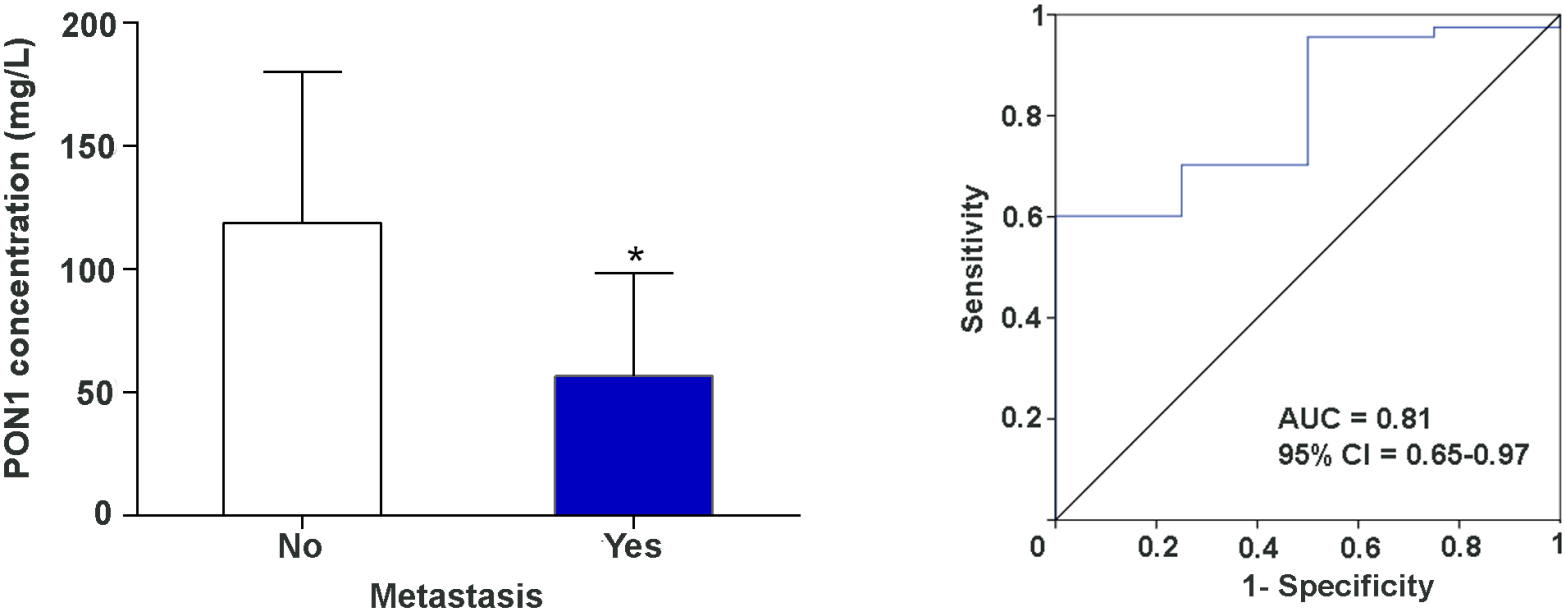
Serum PON1 concentrations post-radiotherapy (RT) in patients with breast cancer with or without metastasis (left panel). Receiver operating characteristics (ROC) plots of serum PON1 concentrations post-RT in breast cancer patients according to whether they had metastasis or not (right panel). AUC: Area under the curve.

We observed significant differences in serum PON1 concentrations pre-RT depending on whether or not the patients were positive for progesterone receptors [88.5 (27.1–215.3) *vs*. 105.9 (38.1–259.1) mg/L, respectively; *P* = 0.029]. Also, the PON1 concentrations post-RT were significantly different between luminal A and luminal B patients [125.7 (16.1–242.5) *vs*. 99.9 (19.6–248.6) mg/L, respectively; *P* = 0.028]. Of note was that serum PON1 concentration post-RT was markedly lower in BC patients with metastases. The AUC of the ROC plot was high, indicating a good sensitivity and specificity of this measurement in distinguishing patients who had metastases from those patients who did not (Fig 2). We did not find any significant associations between the serum PON1 activity or concentration and any other tumor characteristic or toxicological response (Table 5).

**Table 5:** Statistical significance (*P*-value) of the differences between serum PON1 activities and concentrations pre- and post-radiotherapy *vs*. the selected tumor and toxicological variables.

Characteristics	Before radiotherapy		After radiotherapy	
	PON1 activity	PON1 concentration	PON1 activity	PON1 concentration
*Tumor size (TNM system)*	0.964	0.747	0.351	0.559
*Nodes (TNM system)*	0.127	0.821	0.543	0.148
*Metastases (TNM system)*	0.169	0.619	0.323	0.025
*Affected lymph nodes*	0.739	0.637	0.882	0.157
*Histologic grade*	0.183	0.815	0.911	0.859
*Degree of vascular invasion*	0.303	0.664	0.249	0.577
*KI67*	0.329	0.676	0.717	0.347
*Her2*	0.857	0.148	0.554	0.606
*Estrogen receptors*, *negative vs*. *positive*	0.586	0.114	0.980	0.792
*Estrogen receptors*, *%*	0.419	0.233	0.892	0.233
*Progesterone receptors*, *negative vs*. *positive*	0.868	0.029	0.835	0.870
*Progesterone receptors*, *%*	0.713	0.489	0.558	0.351
*Luminal A vs*. *B*	0.923	0.320	0.386	0.028
*Luminal A vs*. *Her2*	0.887	0.421	0.687	0.537
*Luminal A vs*. *triple negative*	0.705	0.561	0.917	0.732
*Luminal B vs*. *Her2*	0.652	0.166	0.289	0.259
*Luminal B vs*. *triple negative*	0.777	0.075	0.331	0.118
*Her2 vs*. *triple negative*	0.719	0.730	0.905	0.893
*Dermatitis*	0.563	0.427	0.097	0.103
*Fibrosis*	0.552	0.492	0.346	0.668
*Disease relapse*	0.720	0.609	0.125	0.174
*Adjuvant chemotherapy*, *yes vs*. *no*	0.785	0.898	0.987	0.969
*Adjuvant hormone therapy*, *yes vs*. *no*	0.314	0.543	0.159	0.646

*P*-value represents the probability that each of the pairs of variables shown in rows and columns are statistically significantly associated. No significant relationship was observed between any of these variables, except for metastases and PON1 concentration post-radiotherapy.

## Discussion

Recently, serum PON1 activity has been reported to be decreased in several types of cancer including BC. The studies, however, remain scarce and have been performed in relatively small numbers of subjects. For example, decreased serum PON1 activity and increased levels of lipid peroxidation have been reported in 45 Pakistani patients with oral squamous cell carcinoma [29], and 30 Turkish patients with esophageal squamous cell carcinoma [30], and a further 25 Turkish patients with papillary thyroid cancer [31]. The activity of PON1 enzyme was shown to be low in 50 Egyptian patients with colorectal cancer but with significant increases one month post-surgery [32]; similar to 49 French patients with BC recurrence [33]. This latter study also showed an inverse relationship between serum PON1 activity and serum amyloid A concentrations. Both these proteins are carried in the circulation bound to HDL particles, and they tend to change in opposite directions in case of inflammation [34]. Conversely, serum PON1 activity has been found to be increased in a group of 23 Turkish patients with prostate cancer, while no changes in lipid peroxidation markers were observed [35]. In the present study, we observed a decrease in serum PON1 activity in BC patients pre-RT, while the serum PON1 concentrations remained unaltered. This latter result suggests that the decrease in the enzyme’s activity is due to enzyme inactivation and not to a decreased PON1 protein synthesis. Diseases involving oxidative stress are associated with decreased serum PON1 activities. This is due to the PON1 active site for lipid peroxide hydrolysis requiring a free sulfhydryl group at cysteine 284; PON1 degrades lipid peroxides by reacting covalently with this site leading to enzyme inactivation [36]. Hence, the net result of increased oxidative stress is decreased PON1 activity with unaltered PON1 concentration, as observed in the present study.

We did not find any significant differences in the frequencies of *PON1*_*192*_ and *PON1*_*55*_ isoforms between BC patients and the control group of subjects. The relationship between *PON1*_*192*_ and *PON1*_*55*_ genes and BC has been recently investigated, but the findings remain equivocal. The R allele of *PON1*_*192*_ polymorphism has been associated with a decreased risk of BC in some studies [11,37,38], while other studies did not find any significant association [13,39–41]. Greater consensus exists with respect to association of the M allele of *PON1*_*55*_ polymorphism and an increased risk of BC; carriers of the M allele of *PON1*_*55*_ polymorphism have lower serum PON1 activity and most studies agree in that the frequency of this allele is higher in BC patients than in the general population [11,13,37–41]. We have not found such an association. Some studies suggest that the relationship between this polymorphism and BC depends on alimentation such as vitamin supplement intake, or medication. Cheng et al. [42] reported an interaction between *PON1*_*55*_ polymorphism and several single nucleotide polymorphisms related to homocysteine and folate metabolism. An explanation could be that the folate cycle regulates the availability of methyl groups through the sequential conversion of methionine to S-adenosylmethionine (SAM), S-adenosylhomocysteine (SAH) and homocysteine and, hence, influences epigenetic modifications of DNA [43]. In addition, Stevens et al. [39] reported that the M allele of *PON1*_*55*_ polymorphism is associated with increased risk of BC only in patients using nonsteroidal anti-inflammatory drugs (NSAIDs). These differences may, perhaps, explain the different findings of the present investigation.

To the best of our knowledge, the present study is the first to report an increase in activity and concentration of serum PON1 in BC patients post-RT. This could be of considerable clinical relevance since PON1 is considered a part of the innate immune system due to its antioxidant, anti-inflammatory and anti-infectious agent properties [44] and, as such, an increase in the activity of PON1 implies an improvement in the general clinical condition of the patient. Our results suggest, then, that the RT benefit for the BC patients could be not only through its direct effect on cancer cells, but also indirectly by improving the organism’s defense mechanisms.

Of further note is the scarcity of studies assessing relationships between the molecular characteristics of BC tumors and the PON1 system. The present study found lower serum PON1 concentrations post-RT in patients belonging to the luminal B subclass. These patients are positive for Her2 and have a high expression of Ki67 membrane antigen. Her2 antigen is a factor in unfavorable prognosis, and its high expression is an indicator of high metastatic liability of the tumor and its possible resistance to anti-hormonal therapy [45]. Similarly, Ki67 is a reliable prognostic marker in determining the number of tumor cells that are in the S phase of the cell cycle. Antigen Ki67 is seen as an independent prognostic factor in relation to tumor recurrence [46,47]. Factors underlying the relationship between PON1 and these antigens are, as yet, unknown. However, recent studies have shown that Her2 and Ki67 expressions are associated with increased expression of various antioxidant enzymes (superoxide dismutase, catalase, glutathione transferase and glutathione reductase) within the tumor cells. This leads to protection of cancer cells against oxidative stress and apoptosis, with subsequent amplification of the proliferative processes [48]. There is a real possibility that PON1 participates in this phenomenon, and the reduced serum concentrations of this protein are a reflection of an inhibited secretion tending towards an increase in the intra-tumor levels of this enzyme. Lykholat et al. [48] suggested that alterations in oxidative status together with higher Her2 and Ki67 expression are indicators of proliferative activity and, as such, could trigger recurrence and metastases in BC patients. In this regard, we observed that serum PON1 concentrations were severely reduced in patients with metastases. Our results are preliminary and should be interpreted with caution because comparisons of 194 patients without *versus* 6 patients with metastases could be considered statistically unreliable. However, we have decided to report these differences since, if confirmed in a wider series of patients, they could have considerable clinical importance. Perhaps the decrease in serum PON1 concentration in patients with metastasis is secondary to an inhibition of its secretion and an increase in its concentration in tissue. As such, the tumor cells would be more protected from death by apoptosis. If this were the case, RT would not be as beneficial in this subgroup of patients. We consider it important that this question is investigated by further studies with a larger number of patients from this subgroup and, in whom, tissue can be made available for the measurement of PON1.

ROC analysis is probably the best, and simplest, way to investigate the diagnostic accuracy of any biochemical biomarker. In this respect, our preliminary results showed a very high analytical sensitivity and specificity for the measurement of post-RT serum PON1 concentration. Indeed, we suggest the implementation of multicenter studies to confirm this finding because, if confirmed, it would indicate that the measurement of serum PON1 concentration post-RT can be a non-invasive and efficient prognostic biomarker of metastasis in BC and, as well, an index of the efficacy of the RT treatment.

We noted, as well, lower serum PON1 concentrations pre-RT in patients positive for progesterone receptors. The relationships between PON1 and the hormone system are largely unknown, and an explanation for this finding cannot be ascertained from the present investigation. Perhaps an explanation derives from tamoxifen treatment in hormone receptor-positive patients. This medication inhibits growth hormone secretion in women [49], and deficiency of this hormone is associated with increased oxidative stress and decreased serum PON1 activity in humans [50]. Hence, it is possible that tamoxifen inhibits PON1 synthesis through its effects on growth hormone. However, this hypothesis is merely speculative.

## Conclusion

The main conclusion of the present study is that the RT benefit for BC patients results not only through its direct effect on cancer cells, but also indirectly by improving the organism’s defense mechanisms. Moreover, our findings, albeit preliminary, suggest that measurement of serum PON1 concentration post-RT could be an efficient prognostic biomarker and an index of the efficacy of the RT. Multicenter, long-term follow-up studies are necessary to ascertain whether PON1 levels (concentrations and/or activities) post-RT can be useful in predicting local relapses and metastases.

## Supporting information

S1 Table (SAV)
